# A Computational Model of Afterimage Rotation in the Peripheral Drift Illusion Based on Retinal ON/OFF Responses

**DOI:** 10.1371/journal.pone.0115464

**Published:** 2014-12-17

**Authors:** Yuichiro Hayashi, Shin Ishii, Hidetoshi Urakubo

**Affiliations:** Department of Systems Science, Graduate School of Informatics, Kyoto University, Yoshida-Honmachi 36-1, Sakyo-ku, Kyoto, 606-8501, Japan; State University of New York Downstate Medical Center, United States of America

## Abstract

Human observers perceive illusory rotations after the disappearance of circularly repeating patches containing dark-to-light luminance. This afterimage rotation is a very powerful phenomenon, but little is known about the mechanisms underlying it. Here, we use a computational model to show that the afterimage rotation can be explained by a combination of fast light adaptation and the physiological architecture of the early visual system, consisting of ON- and OFF-type visual pathways. In this retinal ON/OFF model, the afterimage rotation appeared as a rotation of focus lines of retinal ON/OFF responses. Focus lines rotated clockwise on a light background, but counterclockwise on a dark background. These findings were consistent with the results of psychophysical experiments, which were also performed by us. Additionally, the velocity of the afterimage rotation was comparable with that observed in our psychophysical experiments. These results suggest that the early visual system (including the retina) is responsible for the generation of the afterimage rotation, and that this illusory rotation may be systematically misinterpreted by our high-level visual system.

## Introduction

Motion illusions, a class of visual illusions, are observed when a static image appears to be moving in our visual field [1]. One example is the peripheral drift illusion [2], [3], where illusory motion is perceived when observers gaze or blink in the presence of a particular image in the peripheral visual field [3]. Studies have revealed that the illusory motion observed in the peripheral drift illusion is produced and processed in high-level areas of the visual system such as the primary visual cortex (V1) and the middle temporal visual area (MT) [4], [5]. The peripheral drift illusion is not a purely cognitive phenomenon, but arises from mechanistic characteristics of our visual processing system.

**Figure 1:**
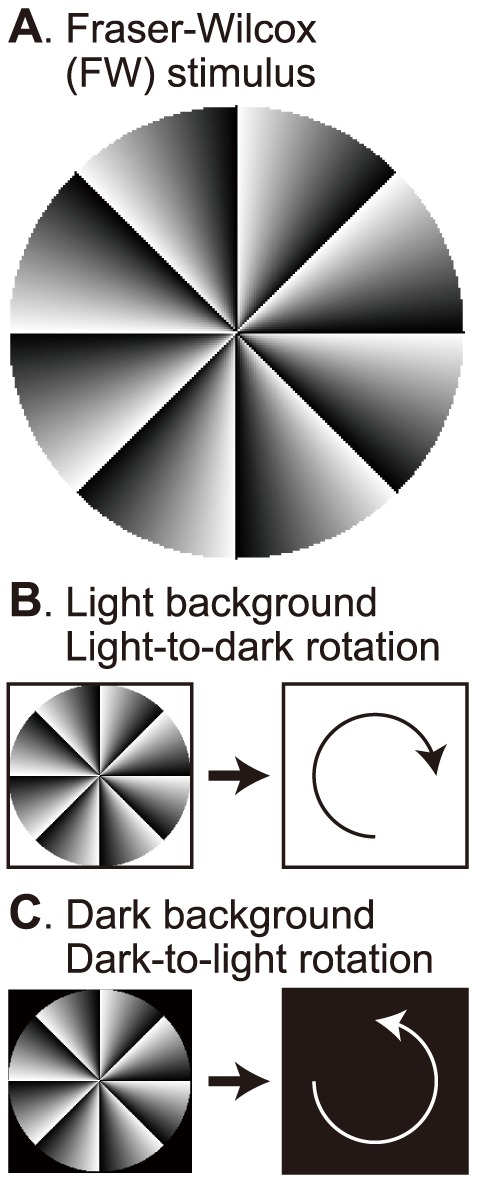
Afterimage rotation in the peripheral drift illusion. (A) The Fraser-Wilcox (FW) stimulus, which was used by Faubert and Herbert [2], [3]. (B) Illusory clockwise (light-to-dark) rotation is seen in the afterimage when the FW stimulus disappears from the light background. (C) Illusory counterclockwise (dark-to-light) rotation in the afterimage is seen on the dark background.

The Fraser–Wilcox (FW) stimulus (Fig. 1A) was used in the original demonstration of the peripheral drift illusion. It consists of circularly repeating patches with sawtooth luminance gradients [2], [3]. The peripheral drift illusion (when induced by presentation of the FW stimulus) occurs at three stages: the eye opening stage (∼0.5 s) [4], [5], the gaze stage [5]–[9], and the afterimage stage, which occurs after eye closing (∼0.7 s) [10]. At the afterimage stage, a prominent clockwise illusory rotation is perceived when the FW stimulus disappears from a light background (Fig. 1B), whereas counterclockwise rotation is perceived when the same stimulus disappears from a dark background (Fig. 1C). According to the light adaptation model, this illusory rotation is caused by the perception of equiluminance locations (lines) in the afterimage that are themselves rotating [4], [10], [11]. After eye closing, the afterimage appears as a compensation of the original FW stimulus and fades out with time. During this process, the equiluminance location moves with time, creating an illusory perception of rotation. This is a reasonable phenomenological description, but a mechanistic explanation of this illusory rotation has not yet been achieved with detailed computational modeling.

In the present study, we provide a mechanistic explanation for the afterimage rotation of the FW stimulus by using a computational model that incorporates physiological knowledge of the early visual system. This model is able to account for the results of two psychophysical experiments that were also run in the present study. Our model was based on a light adaptation model so as to reproduce fast processing of retinal afterimages [9], and incorporated the ON-type and OFF-type visual pathways in the early visual system (especially in the retina). We found that not only did our model successfully reproduce the afterimage rotation, but also that the direction and velocity of the afterimage rotation in our model were quantitatively consistent with those obtained in our psychophysical experiments. This consistency between our computational model and findings in human psychophysical experiments suggests that our model-based analysis provides a more systematic interpretation of the peripheral drift illusion than the simple light adaptation model.

## Results

**Figure 2:**
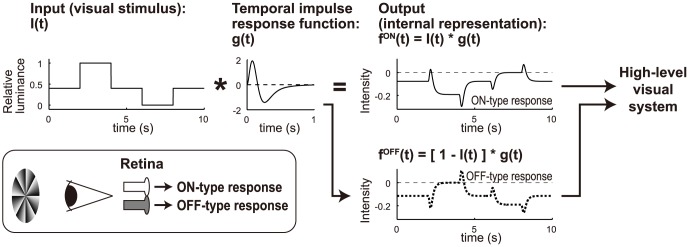
Retinal ON/OFF model. A visual time-series stimulus (*I*(*t*)) was assigned to a certain receptive field of the retina. The retinal units (neurons) included in the receptive field, which have a biphasic temporal impulse response (TIR) function (*g*(*t*)), produced two types of time-series responses: ON- and OFF-type (*f^ON^* (*t*) and *f^OFF^*(*t*), respectively). These responses are assumed to be integrated at higher levels of the visual system.

We adopted a computational model to explain the afterimage rotation of the FW stimulus (Fig. 1). The afterimage rotation is known to end within one second of the disappearance of the FW stimulus [10], and this period is compatible with the fast light adaptation of the visual system [12], [13]. A previous psychophysical experiment found that the peripheral drift illusion was associated with the biphasic shape with a large undershoot of the temporal impulse response (TIR) function [9]. The TIR function is an approximation of the fast light adaptation (Fig. 2A). This large undershoot may be a cause of the afterimage rotation, which motivated us to test a linear light adaptation model with a biphasic TIR function (S1A Figure). We applied the linear light adaptation model to the FW stimulus but did not observe any afterimage rotation in the model's output, even with a large undershoot (S1A Figure). Since this discrepancy might have arisen from nonlinearity in the light adaptation model, we next tested a nonlinear light adaptation model [14]. Three types of nonlinear adaptation mechanisms were introduced to this model: divisive light adaptation, subtractive light adaptation, and contrast gain control (S1B Figure) [14]. However, this nonlinear model was also unable to elicit the afterimage rotation of the FW stimulus (S1B Figure). Taken together, these simple light adaptation models themselves could not explain the afterimage rotation of the FW stimulus, implying that some missing mechanism is necessary.

**Figure 3:**
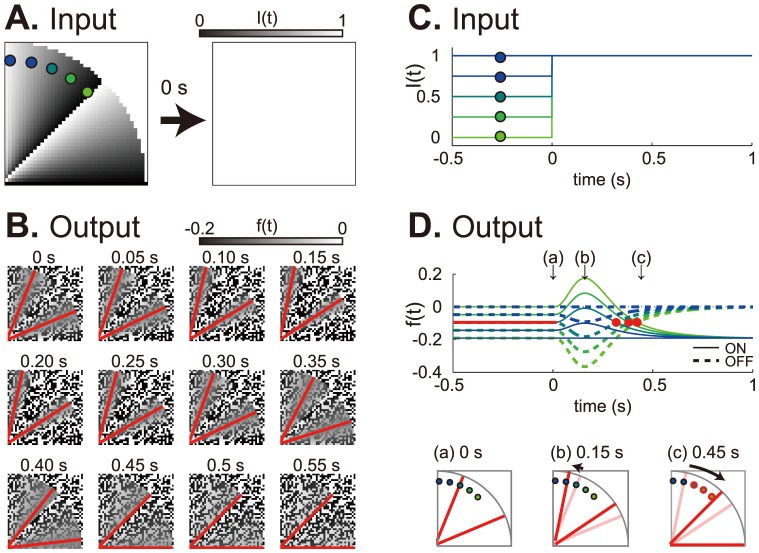
The retinal ON/OFF model produced afterimage rotation of the FW stimulus on the light background. (A) Input. A quarter of the FW stimulus suddenly disappeared and was replaced by the light background at 0 s. (B) Output after the disappearance of the FW stimulus. Red lines indicate the focus lines for which the ON- and OFF-type responses showed comparable values. (C) Time courses of input. Each marked line corresponds to the input time-series (in terms of the luminance level) at the marked position in (A). (D) Time courses of outputs from the ON- and OFF-type units (top). Each colored line represents the output in response to the input with the same color as in (C). Red lines and points indicate the focus lines and points. Schematic drawings of outputs at 0 s (a), 0.15 s (b) and 0.45 s (c) (bottom). Focus lines rotated slightly counterclockwise from 0 s (thin red lines) to 0.15 s (red lines) (b), then prominently rotated clockwise from 0.15 s (thin red lines) to 0.45 s (red lines) (c). The red marked points in (c) correspond to the three red points in the top panel.

**Figure 4:**
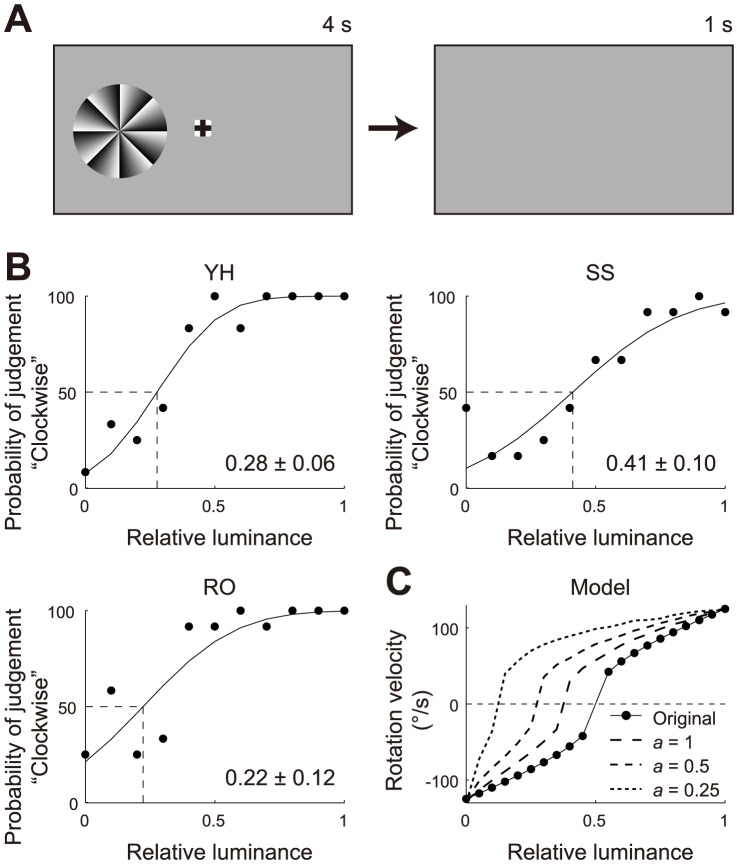
The direction of the afterimage rotation was examined by varying the background luminance. (A) Diagram of the human psychophysical experiment. In the gaze period (4 s), the FW stimulus and the fixation cross were both presented on the background with a specific luminance. After the gaze period, both the FW stimulus and the fixation cross disappeared, but the background was not changed. The participants were instructed not to move their eyes during this afterimage period (1 s). (B) Probability of seeing clockwise rotation (by the three observers: Y.H., S.S., and R.O.). Each psychometric curve was individually fitted with a cumulative Gaussian function by means of a least squares method. The means (μ) of the Gaussian functions are given with their 95% confidence intervals. (C) Representative rotation velocity of the retinal ON/OFF model when changing the background luminance (original). Those from the modified model based on Weber's law were also plotted (*a* = 1, *a* = 0.5 and *a* = 0.25, dashed lines). The relative luminance levels that gave a zero-rotation velocity were 0.38, 0.27 and 0.12 for *a* = 1, *a* = 0.5 and *a* = 0.25 respectively, and 0.5 for the original.

In the early visual system, and in the retina in particular, there are two distinct visual pathways: ON-type and OFF-type pathways that produce ON-type and OFF-type responses to visual stimulation (Fig. 2) [15]. The signals mediated by the two pathways are integrated into the higher-level visual system, which leads to visual perception. We incorporated the existence of ON- and OFF-type units (neurons), into a linear adaptation model. Hereafter, this model is called the retinal ON/OFF model. When the FW stimulus was presented, the retinal ON/OFF model produced the focus lines, for which the ON- and OFF-type units showed the most comparable responses in the visual field (Fig. 3B). When the FW stimulus disappeared from the light (white) background, these focus lines first rotated in the counterclockwise direction slightly, then rotated in the clockwise direction prominently (Fig. 3BD; S1 Video). This prominent clockwise rotation was similar to that perceived by human observers (Fig. 4B) [4], [10]. By continuously presenting the FW stimulus, the output of each unit in the retinal ON/OFF model reached a steady state that depended on input luminance and hence on the location in the visual field (before 0 s in Fig. 3D). Because of the large undershoot of the TIR function (Fig. 2), the disappearance of the FW stimulus triggered a large undershoot of the responses of the ON-type units as well as a large overshoot of those of the OFF-type units (after 0 s in Fig. 3D). The disappearance of the dark part from the light background produced a larger undershoot and overshoot (greener lines, Fig. 3D), whereas the disappearance of the light part had smaller effects (bluer lines, Fig. 3D). The larger undershoot and overshoot caused crossover between the ON units' recovery from the overshoot and the OFF units' recovery from the undershoot with a long latency (greener lines, Fig. 3D), while the smaller ones led to a short latency (bluer lines, Fig. 3D). This luminance-dependent latency of crossing (focusing) of retinal ON and OFF responses (red points, Fig. 3D) caused the afterimage rotation of focus lines in the FW stimulus (Fig. 3B).

When the FW stimulus was presented on the light background, the retinal ON/OFF model demonstrated prominent clockwise rotation in the afterimage. We further found that the focus lines mainly rotated counterclockwise on the dark (black) background (S2 Figure; S2 Video), as was perceived by the human observers [4], [10]. The retinal ON/OFF model's dependence on background luminance motivated us to perform a human psychophysical experiment that examined the direction of the afterimage rotation when varying the background luminance (Experiment 1 in Methods; Fig. 4A; S1 Table). As the background luminance increased, so did the probability of seeing the clockwise rotation (Fig. 4B). When the relative luminance was set at 0.28±0.06, 0.41±0.10, and 0.22±0.12 for the three observers (Y.H., S.S., and R.O., respectively), the rates of seeing clockwise and counterclockwise rotations were balanced (Fig. 4B). Interestingly, when the same experiment was performed using our retinal ON/OFF model, the model showed a similar dependence on the background luminance (solid line, Fig. 4C). The direction of the model's rotation was balanced when the relative background luminance was set at 0.5. This balanced luminance level was slightly higher than those in the psychophysical experiment (0.22–0.41; Fig. 4B). However, this discrepancy could be reconciled if the units in the retinal ON/OFF model responded logarithmically to the luminance level, based on Weber's law [12] (dashed lines, Fig. 4C; Methods). With an applied logarithmic scale factor of *a* = 1, *a* = 0.5 and *a* = 0.25, the retinal ON/OFF model resulted in a balanced luminance level of 0.38, 0.27 and 0.12 respectively (Fig 4C), which led to a zero-rotation velocity. These balanced luminance levels were comparable with those observed in the psychophysical experiment. Since the logarithmic function is increasing and concave, the balanced luminance levels of the modified model were less than 0.5 for any scale factor of *a*, accounting for the biased sensitivity to darker inputs. Accordingly, the retinal ON/OFF model could successfully reproduce the background luminance dependence of the afterimage rotation observed in human participants.

**Figure 5:**
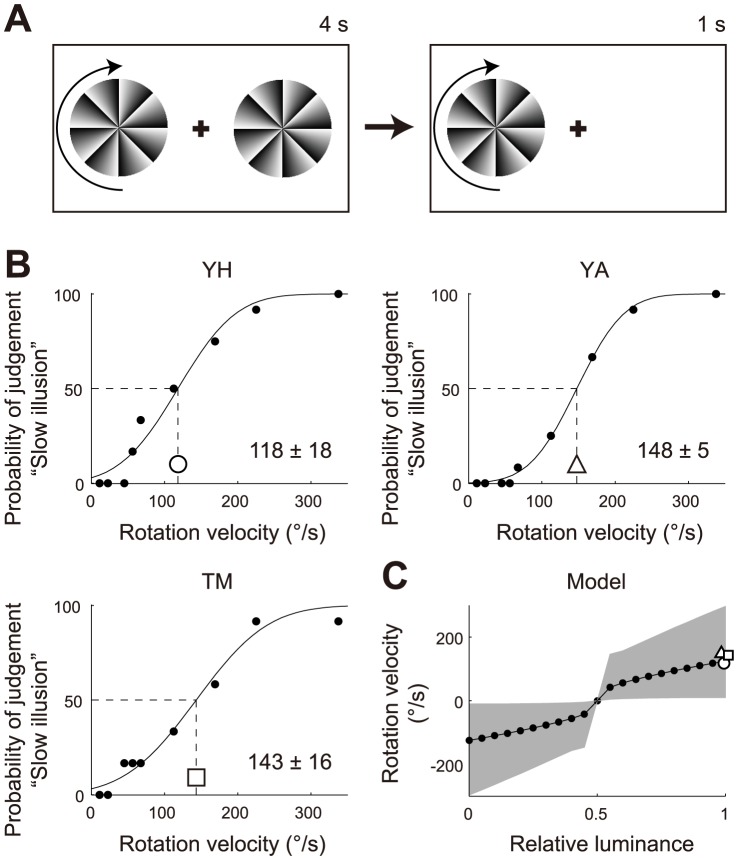
Velocities of the afterimage rotation on the light background. (A) Diagram of the human psychophysical experiment. In the gaze period (4 s), the still image of the FW stimulus, the reference rotating FW stimulus, and the fixation cross were presented on the light background, on which the locations of the still and reference FW stimuli were randomly allocated to the left or right. After the gaze period, the still FW stimulus disappeared, but the reference stimulus and the fixation cross remained. The observers were instructed to fixate on the fixation cross during this afterimage period (1 s) and then to report whether the perceived velocity of the afterimage of the absent FW stimulus was faster or slower than that of the reference stimulus. (B) Probability of seeing slower afterimage rotation than the reference rotation (the three observers: Y.H., Y.A., and T.M.). Each psychometric curve was fitted individually with a cumulative Gaussian function by means of a least squares method. The means (μ) of the Gaussian functions are given with their 95% confidence intervals. (C) Representative rotation velocity of the retinal ON/OFF model when changing the background luminance. The profile of the rotation velocity, which increased with time, is shown as a grey area. Three marks indicate the rotation velocities obtained in the psychophysical experiment (marks correspond to those in (B)).

To further validate the retinal ON/OFF model, we performed another psychophysical experiment to quantify the perceived velocity of the afterimage rotation (Experiment 2 in Methods; Fig. 5A; S2 Table) [5], [9]. The velocity of the afterimage rotation was determined as 118±18°/s, 148±5°/s, and 143±16°/s for the three observers (Y.H., Y.A., and T.M., respectively; Fig. 5B). All observers reported that the afterimage rotation was consistent over locations; i.e., the whole FW image coherently rotated around its center. In the retinal ON/OFF model, just after the stimulus disappearance, the focus lines slightly rotated in one direction, and after 150–200 ms, they turned to rotate prominently in the opposite direction. The velocity of the latter rotation increased over time (grey area in Fig. 5C). The representative rotation velocity on the light background was 125°/s, comparable with those of the human observers (Fig. 5C). Although this velocity was obtained by setting the time constants at values around five times slower (∼200 ms) than those in the previous study that quantified the TIR function [9], the slow time constants have a certain rationale. The fast light adaptation to stepwise inputs is known to be slower than to impulse inputs [16], and the slow time constant is consistent with the recovery time constant of the nonlinear adaptation model (see the left and right panels of S1D Figure) [14]. Accordingly, our retinal ON/OFF model performs well in reproducing the velocity of the afterimage rotation of the FW stimulus observed in human participants.

## Discussion

**Figure 6:**
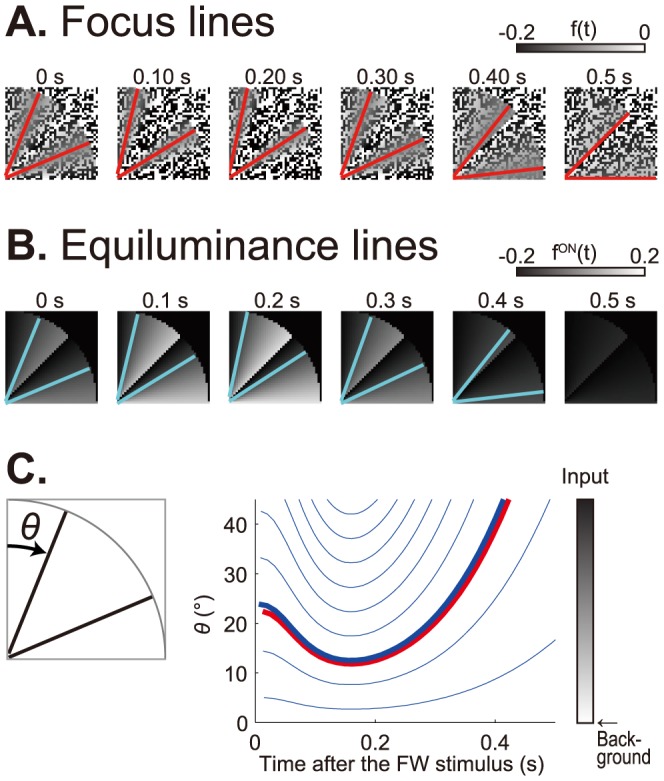
The focus lines correspond to specific equiluminance lines in the light adaptation model, which are characterized by small standard deviations in the performance of the retinal ON/OFF model. Inputs to both the retinal ON/OFF model and the light adaptation model were the same (Fig. 3(A)) on the light background. (A) Outputs of the retinal ON/OFF model. Red lines indicate the focus lines, which were accompanied by small standard deviations of the ON- and OFF-type responses. (B) Output of the light adaptation model 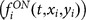. Cyan lines represent typical equiluminance lines. (C) Time development of the angles (rotations) of the focus line (red line) and equiluminance lines (blue lines, for different equiluminance levels) where the angle *θ* was specified in the left panel. The thick blue line corresponds with the example equiluminance lines in (C).

Here we presented the retinal ON/OFF model, which is based on fast light adaptation and the retinal architecture consisting of ON- and OFF-type neurons. This model successfully reproduced the afterimage rotation of the FW stimulus, which was mechanistically represented by the rotation of focus lines. The mechanism of the focus line rotation is closely related to the conventional mechanism of the afterimage rotation: equiluminance rotation in the afterimage (Fig. 6) [4], [10]. When we defined a light adaptation model which output only the ON responses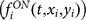, we could trace the rotation of the lines that gave a uniform output level, i.e., equiluminance lines (Fig. 6B). Some of these equiluminance lines rotated in the afterimage (blue lines in Fig. 6C) [4], [10], but this equiluminance rotation depended on the input luminance. When the input luminance was close to the background luminance, the equiluminance rotation became too slow (*θ*∼0° in Fig. 6C). However, if the input luminance was far away from the background luminance, the one-way equiluminance rotation did not occur (*θ*∼45° in Fig. 6C). Thus, there is location dependency in the velocity of the afterimage rotation, according to the simple light adaptation model. In contrast, the focus line in the retinal ON/OFF model can be seen as a special equiluminance line (red line in Fig. 6C) in the light adaptation model. The standard deviations of amplitudes of the retinal ON- and OFF-type responses were small around the focus lines, and the areas with small standard deviations also rotated in accordance with the focus line rotation (Fig. 3B). Although the focus line is thus a pickup from multiple equiluminance lines, its rotation velocity showed good consistency with those in human psychophysical experiments. It is important to note that human observers reported location-independent (*θ*-independent) rotation velocities rather than location-dependent rotation velocities in our psychophysical experiments. These observations would lead to different interpretations of the afterimage rotation in the peripheral drift illusion. In the retinal ON/OFF model, the afterimage in its entirety should be seen to rotate at a uniform velocity, as reported by the human observers, but it instead shows location dependency in the light adaptation model. If we do not assume a complicated decoding mechanism to remove location dependency in the high-level visual system, the focus line would be a simpler and more robust representation than the set of equiluminance lines in the light adaptation model. Our retinal ON/OFF model can thus provide a more robust scenario to the afterimage rotation in the peripheral drift illusion than the simple light adaptation model.

Light adaptation has been modeled based on psychophysical experiments [17]–[19], and some modeling studies have aimed at linking psychophysical experiments with retinal physiology [13], [14]. Indeed, many characteristics of light adaptation can be explained by modeling the functions of retinal ganglion cells and other retinal mechanisms. As one prominent feature of the early visual system is the existence of ON- and OFF-type pathways [20], it is reasonable to develop a model that combines the fast light adaptation and the retinal architecture equipped with ON- and OFF-type units.

ON- and OFF-type responses in the early visual system are integrated into areas of the high-level visual system such as the primary visual cortex (V1) [15], [21]. Generally, neurons in the high-level visual system have larger receptive fields than those in the early visual system, so as to integrate the input of lower-level neurons, enabling the capture of global features of input images. If this integration process was represented by a simple spatial smoothing filter, the high-level neurons would not be able to distinguish the illusory afterimage rotation from the actual rotation, because they share the same activity patterns with the low-level neurons. When the actually rotating images of the FW stimulus were used in the retinal ON/OFF model, the model also produced the focus rotation (S3 Figure). Thus, the focus rotation could be interpreted as a signal to be processed in the high-level visual system, and this may cause misinterpretations which could manifest as motion illusions in our visual perception.

The peripheral drift illusion at the gaze stage has been extensively investigated from both psychophysical and neurophysiological viewpoints, with a particular interest in Kitaoka's rotating snakes illusion [22]. Previous studies have shown that the peripheral drift illusion at the gaze stage is triggered by saccadic eye movements [7], [23]–[25]. Other studies have suggested that saccadic eye movements lead to retinal image slip, and that the error compensation of the retinal image slip may induce the illusory rotation [8], [25], [26], which can be modeled by an optical flow algorithm [8] or a spatiotemporal energy filter [26]. Researchers have also argued that the peripheral drift illusion after eye opening may be a consequence of the contrast dependent latency of visual processing [6], or compound adaptation, which could drive both contrast- and luminance-dependent illusions [5]. Consistent with all lines of evidence and modeling is that the illusory rotation requires the resetting or refreshing of retinal images. The reset or refresh process involves the sudden appearance and disappearance of images, which cause the transient undershoot and overshoot of the fast light adaptation, i.e., afterimages. Thus, the afterimages and focus rotation should contribute not only to the afterimage stage but also to the other stages that lead to the peripheral drift illusion, including the eye-opening and gaze stages. Indeed, the illusory rotation by the second FW stimulus is interfered if the first and second FW stimuli are repetitively presented at a short inter-stimulus interval [27], implying the contribution of afterimages to this illusion. This discussion suggests that our retinal ON/OFF model may provide a systematic framework to understand the mechanisms underlying the peripheral drift illusion at all of its stages. Furthermore, our computational modeling based on physiological knowledge of the early visual system may be able to uncover the common mechanisms underlying different types of visual illusions which are based on fast light adaptation or afterimages [28].

The peripheral drift illusion results from the presentation of types of visual stimuli other than the FW stimulus. A typical example is the Kitaoka–Ashida stimulus, which consists of segmented luminance steps in radially cut sectors (S4D Figure) [22]. When the Kitaoka–Ashida stimulus was applied to the retinal ON/OFF model, however, there was no focus line rotation (S4D Figure, S5 Video). This discrepancy between the psychophysics and model simulation arose because of the stepwise luminance changes in the Kitaoka–Ashida stimulus. Spatial stepwise changes in luminance not only activate the edge detection mechanism of the early visual system [15], [29], [30], which is not incorporated in the current retinal ON/OFF model, but also cause other types of illusion such as perceptual filling in [31]–[33], which appears also in the afterimage [34]. The peripheral drift illusion induced by spatial stepwise changes in luminance as in the Kitaoka–Ashida stimulus would be a consequence of a combination of multiple mechanisms, and is thus not directly addressed by the retinal ON/OFF model. Interestingly, the retinal ON/OFF model can simulate the afterimage rotation if the spatial stepwise changes (edges) of luminance are simply blurred (S4BE Figure). In fact, focus line rotation appeared in the afterimage of a Kitaoka–Ashida stimulus that had been spatially blurred by a Gaussian filter (S4E Figure, S6 Video) [9].

Another interesting point of the retinal ON/OFF model is that it can predict the image that produces the strongest afterimage rotation with constant velocity, without relying on psychophysical experiments. The predicted luminance profile (S4F Figure, S7 Video) is similar to the existent luminance profile called “Sakura”, which actually induces consistent illusory rotation [26]. This ‘optimal’ luminance profile has a relatively large light area, and such a characteristic may have enhanced our misperception in the afterimage.

Since our model incorporates only the architecture of the early visual system [15], [21], it does not provide by itself any solid solution to the neural basis by which the higher-level visual system “sees” the focus line rotation in the afterimage [35]. Our retinal ON/OFF model, however, provides a more systematic interpretation of the peripheral drift illusion than the simple light adaptation model, in the following three ways. First, the focus line of the retinal ON/OFF model is a pickup from multiple equiluminance lines of the light adaptation model [4], [10], [11] (Fig. 6C), and there is thus no need to assume the process in which the high-level visual system picks up the prominent rotating line from multiple equiluminance lines for our perception. Second, in the retinal ON/OFF model, the afterimage in its entirety rotates at a uniform velocity as reported by human observers. Third, the retinal ON/OFF model produces the focus line rotation when the actually rotating FW stimulus is applied (S3 Figure). These advantages suggest the important contribution of the physiologically characteristic architecture inherent to the early visual system to the peripheral drift illusion. Thus, the development of the retinal ON/OFF model can be considered a step to bridge the phenomenological and mechanistic aspects of the peripheral drift illusion, a prominent example of systematic misinterpretations of our visual system.

## Materials and Methods

### Retinal ON/OFF model

Visual light adaptation has been described by a TIR function as a first-order approximation [19]. In our retinal ON/OFF model, time-varying visual images *I*(*t*, *x_i_*, *y_i_*) are linearly transformed into an internal representation *f_i_*(*t*, *x_i_*, *y_i_*), being convolved with a TIR function *g*(*t*):



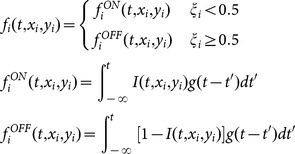
where *t* is the time (in seconds) and *i* is the index that indicates a unit (neuron) located at (*x_i_*, *y_i_*) in the visual field. Here, *ξ_i_* denotes either ON-type (when positive) or OFF-type (when negative), which was drawn from the uniform distribution on the interval [0,1]; each unit was assigned as ON- or OFF-type and the type was fixed along the time. These ON- and OFF-type units are assumed to be placed densely, in comparison to the pixel resolutions of the input images, in the visual field, and constitute the retinal ON/OFF model. Furthermore, the TIR function *g*(*t*) is given by



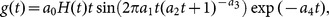
where the Heaviside function is given by *H*(*t*) = 1 for *t*≥0 and 0 otherwise. The constants *a*_0_, *a*_1_, *a*_2_, *a*_3_, and *a*_4_ in the TIR function are dependent on environmental and physiological properties such as background luminance, retinal eccentricity, and observers [16], [19], [36]. A previous study that demonstrated the importance of biphasic and negatively-biased TIR for the peripheral drift illusion at the gaze stage set the TIR constants at (*a*_0_, *a*_1_, *a*_2_, *a*_3_, *a*_4_) = (229.5, 21.09, 1, 9.96, 39.05), based on a psychophysical experiment [9]. We used different settings for the constants, (*a*_0_, *a*_1_, *a*_2_, *a*_3_, *a*_4_) = (45.9, 4.2, 0.2, 9.4, 7.8), which were five times slower than those used in the previous study [9]. Using the retinal ON/OFF model with these settings for the TIR function, we attempted to reproduce the velocity of afterimage rotation obtained in the psychophysical experiment (Fig. 4).

### Modified model

In the modified retinal ON/OFF model, the input image was assumed to be transformed into the logarithmic scale according to Weber's law [12]:



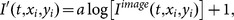
where *I^image^* (*t*, *x*, *y*) is the luminance intensity of the input images, which is assumed to span within [exp(−1/*a*), 1] from dark to light, and *a* is the logarithmic scale component. We tested three cases of *a* = 1, 0.5, 0.25 (Fig. 4C). In the modified model, the transformed image *I'*(*t*, *x*, *y*), which spans within [0, 1], was input to the original retinal ON/OFF model.

### Focus point and rotation velocity

Since the ON- and OFF-type units are densely placed in the retinal ON/OFF model, we can approximate that at a particular location (*x_i_*, *y_i_*) in the visual field, ON- and OFF-type responses at time *t* are given by 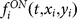 and 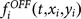, respectively. Under this assumption, the ‘focus’ points at time *t* were defined as the set of location (*x_i_*, *y_i_*) at which the ON- and OFF-type responses gave the locally most similar values, i.e., 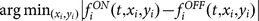 (Figs. 3BD, 6, S2BD, S3BD and S4 Figures). Because the image intensity *I*(*x*, *y*) provided by the FW stimulus to the retinal ON/OFF model was the same if the location (*x*, *y*) was of the same central angle, and moreover because the TIR function did not include stochastic factors, the focus points comprised a couple of radial lines (Figs. 3B, 6, S2B, S3B and S4 Figures).

When the FW stimulus disappeared from the light or dark background, the focus lines moved. Just after the onset of the stimulus disappearance, the focus lines slightly rotated in one direction, but after 150–200 ms from the stimulus disappearance, they turned to rotate prominently in the opposite direction (Fig. 3BD; S2 Figure). To quantify the speed of this latter rotation, we measured the time-series of the focus lines in terms of central angles, and then calculated the average angular velocity (°/s) by linear regression, using this as the representative rotation velocity (Figs. 4C and 5C). We also superimposed the whole range of the increasing rotation velocity from its start (∼0°/s) to end (absolute maximum velocity; grey area in Fig. 5C).

### Psychophysical experiments

#### Participants

In both psychophysical experiments, the first author (Y.H.) and two naïve observers per experiment took part. All were 21–23 years old males and had normal or corrected-to-normal vision. The experiments were approved by the Ethics Committee of Advanced Telecommunications Research Institute International (ATR), Japan, and all participants gave written informed consent before the experiments and their aims were explained to them. The total testing time for each participant was roughly 40 minutes for Experiment 1 and 30 minutes for Experiment 2.

#### Apparatus

The visual stimuli were presented on a 21-inch CRT monitor (IBM C220P, 1280×1024 pixels, refresh rate 75 Hz) controlled by a computer. Observers were instructed to relax and to fix their head on the headrest of a dental chair 60 cm from the display. The monitor size was 45°×33°, where 1° corresponds to one degree of visual angle.

#### Stimulus

We presented a circular stimulus whose eight sectors were gradually graded from dark (black) to light (white) in the counterclockwise direction (Fig. 1A). The luminance ranged from 0 cd/m^2^ (dark, black) to 94 cd/m^2^ (light, white). The diameter of the circle was 15 degrees. A fixation cross was presented in the center of the monitor. The distance between the fixation cross and the stimulus center was 11 degrees.

#### Experiment 1 Procedure

We broadly followed the procedure of previous experiments [5], [9]. At the beginning of each trial, a fixation cross was presented on the background with a specific luminance for 7 s (fixation period). Then, the FW stimulus was presented randomly on either the left or right side of the fixation cross for 4 s (gaze period, Fig. 3A). After the gaze period, the FW stimulus and the fixation cross disappeared, leaving only the background luminance, which was set to the same level as in the fixation period (Fig. 3A). During this afterimage period of 1 s, observers were requested not to move their eyes. After the afterimage period, the background was replaced by the light background and the observers were asked to make a forced-choice judgment as to whether the FW stimulus in the afterimage seemed to rotate clockwise or counterclockwise. Immediately after the observer gave a response, the next trial started. In each trial, the background luminance was selected from 11 linearly-spaced intensities (from dark to light). For each background luminance, 12 responses were collected. The order of presentation of the background luminance was individually randomized to eliminate possible history dependence across the trials. The psychometric curve for each participant was fitted with a cumulative Gaussian function by applying the least-squares method to 132 ( = 11×12) data points.

#### Experiment 2 Procedure

First, the dark (black) background was presented for 7 s. Then, a fixation cross was presented on the light (white) background for 7 s (fixation period). After the fixation period, two FW stimuli were presented on both sides of the fixation cross (gaze period in Fig. 4A), for 4 s. One of them (randomly selected) was a still image of the FW stimulus, and the other was the same image, but rotating clockwise at a certain speed. After the gaze period, the still FW stimulus disappeared, leaving the fixation cross and the rotating FW stimulus (Fig. 4A). This afterimage period continued for 1 s. After the afterimage period, the fixation cross and the rotating FW stimulus were replaced by the light background, and then the observers were asked to make a forced-choice judgment as to which FW stimulus in the afterimage period (one actually rotating, and the other seemingly rotating) seemed to rotate more rapidly. Immediately after the observer gave a response, the next trial started. In each trial, the rotation speed of the rotating FW stimulus was randomly selected from nine predefined parameters (11.3°/s, 22.5°/s, 45°/s, 56.3°/s, 67.5°/s, 112.5°/s, 168.8°/s, 225°/s, 337.5°/s), where 1°/s corresponds to one degree of polar angle per second. Twelve responses were collected for each rotation speed and for each participant. The order of presentation of the rotating FW stimuli was randomized. The psychometric curve for each participant was fitted with a cumulative Gaussian function by applying the least-squares method to the 72 ( = 6×12) data points. At the end of the experiment, the participants were asked questions about how they perceived the afterimage rotation, especially in terms of its spatial coherency.

## Supporting Information

S1 FigureAdaptation models did not show afterimage rotation. (A) Input. One quarter of the FW stimulus disappeared from the light (white) background. (B) Outputs of a linear adaptation model (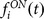; left) and a nonlinear adaptation model (right) [14]. (C) Time courses of inputs to the models. Each marked line corresponds to the input time-series (in terms of the luminance level) at the marked position in (A). Note that the input to the nonlinear adaptation model is specified not by luminance, but by illuminance. Here, we set the dark illuminance as *I* = 30Td and the light illuminance as *I* = 3000Td. Three-thousand Td (illuminance) corresponds to 104 cd/m^2^ (luminance) with a pupil diameter of 6 mm. (D) Time courses of outputs from the models. Each colored line represents the output in response to the input with the same color as in (C).(TIF)

S2 Figure The retinal ON/OFF model produced an afterimage rotation of the FW stimulus on the dark background. (A) Input. One quarter of the FW stimulus suddenly disappeared and was replaced by the dark background at 0 s. (B) Outputs after the disappearance of the FW stimulus. Red lines indicate the focus lines on which the ON- and OFF-type responses, *f^ON^*(*t*) and *f^OFF^*(*t*) in Fig. 2, respectively, showed comparable values. (C) Time courses of inputs. Each marked line corresponds to the input time-series (in terms of the luminance level) at the marked position in (A). (D) Time courses of outputs from the ON- and OFF-type units (top). Each colored line corresponds to the output in response to the input with the same color in (C). Red lines and points indicate the focus lines and points. Schematic drawing of outputs at 0 s (a), 0.15 s (b) and 0.45 s (c) (bottom). Focus lines slightly rotated counterclockwise from 0 s (thin red lines) to 0.15 s (red lines) (b), then prominently rotated clockwise from 0.15 s (thin red lines) to 0.45 s (red lines) (c). The red marked points in (c) correspond to the three red points in the top panel.
(TIF)

S3 Figure Focus rotation of ON- and OFF-type responses compared with actual rotation of the FW stimulus. (A) Input. The FW stimulus rotated clockwise with an angular velocity of 125°/s. (B) Output. Red and orange lines indicate focus lines on both of which the ON- and OFF-type responses showed comparable values. The number of focus lines was doubled from that in Fig. 2, because the focus line rotation appeared not only at the timing of the disappearance, but also at the timing of the appearance of the FW stimulus. Note that the appearance and disappearance occurred at each location, suggesting that it was elicited by the actually rotating FW stimulus. (C) Time courses of inputs. Each marked line corresponds to the input time-series at the marked position in (A). (D) Time courses of outputs from the ON- and OFF-type units. Each colored line represents the output in response to the input with the same color as in (C). Red and orange points indicate focus points.
(TIF)

S4 Figure Focus line rotation of ON- and OFF-type responses to various types of visual stimuli: (A) the FW stimulus, (B) the blurred FW stimulus (Gaussian filter: σ = 1.35°), (C) alternating dark and light cut sectors, (D) the Kitaoka–Ashida stimulus [22], (E) the blurred Kitaoka–Ashida stimulus (Gaussian filter: σ = 1.35°), and (F) the optimal luminance profile. The first column shows the input image on the light background. The second column shows the output from the retinal ON/OFF model at 0 s where the red lines indicate the focus lines of the ON- and OFF-type responses (just for the top right quarter of the whole output). The third column shows the luminance profile of the input image, and the fourth column shows the time development of the angles of the focus lines (red lines).
(JPG)

S1 Table Data points of Fig. 4B and results of experiment 1. Probability of seeing clockwise rotation by the three observers (Y.H., S.S., and R.O.). In each trial, the background luminance was randomly selected from 11 linearly-spaced intensities.
(PDF)

S2 Table Data points of Fig. 5B and results of experiment 2. Probability of seeing slower afterimage rotation than the reference rotation by the three observers (Y.H., Y.A., and T.M.). In each trial, the rotation speed of the rotating FW stimulus was randomly selected from nine predefined parameters (11.3°/s, 22.5°/s, 45°/s, 56.3°/s, 67.5°/s, 112.5°/s, 168.8°/s, 225°/s, 337.5°/s).
(PDF)

S1 Video The retinal ON/OFF model produced afterimage rotation of the FW stimulus on a light background.
(MP4)

S2 Video The retinal ON/OFF model produced afterimage rotation of the FW stimulus on a dark background.
(MP4)

S3 Video Output of the retinal ON/OFF model in response to the blurred FW stimulus on a light background S4B Figure).
(MP4)

S4 Video Output of the retinal ON/OFF model in response to the alternating dark and light cut sectors on a light background (S4C Figure).
(MP4)

S5 Video Output of the retinal ON/OFF model in response to the Kitaoka–Ashida stimulus on a light background (S4D Figure).
(MP4)

S6 Video Output of the retinal ON/OFF model in response to the blurred Kitaoka–Ashida stimulus on a light background (S4E Figure).
(MP4)

S7 Video Output of the retinal ON/OFF model in response to the optimal luminance profile on a light background (S4F Figure).
(MP4)
