# Supplementary figures and images for: A Computational Model of Afterimage Rotation in the Peripheral Drift Illusion Based on Retinal ON/OFF Responses
Source: PLoS One. 2014 Dec 17;9(12):e115464. doi: 10.1371/journal.pone.0115464 (PMC4269430; doi:10.1371/journal.pone.0115464)

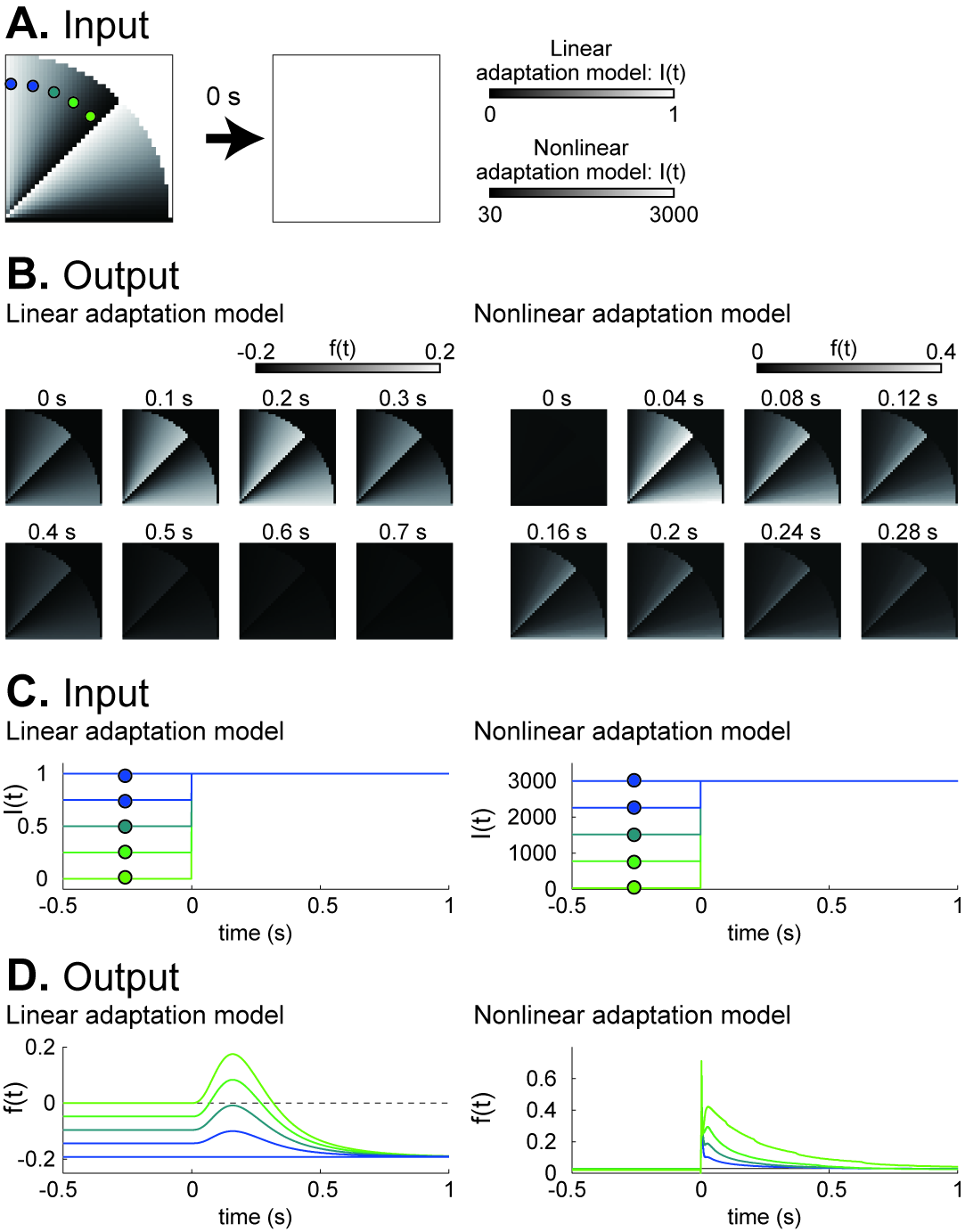

Supplement: S1 Figure — Adaptation models did not show afterimage rotation. (A) Input. One quarter of the FW stimulus disappeared from the light (white) background. (B) Outputs of a linear adaptation model (; left) and a nonlinear adaptation model (right) [14]. (C) Time courses of inputs to the models. Each marked line corresponds to the input time-series (in terms of the luminance level) at the marked position in (A). Note that the input to the nonlinear adaptation model is specified not by luminance, but by illuminance. Here, we set the dark illuminance as I = 30Td and the light illuminance as I = 3000Td. Three-thousand Td (illuminance) corresponds to 104 cd/m2 (luminance) with a pupil diameter of 6 mm. (D) Time courses of outputs from the models. Each colored line represents the output in response to the input with the same color as in (C). (TIF) [file pone.0115464.s001.tif]

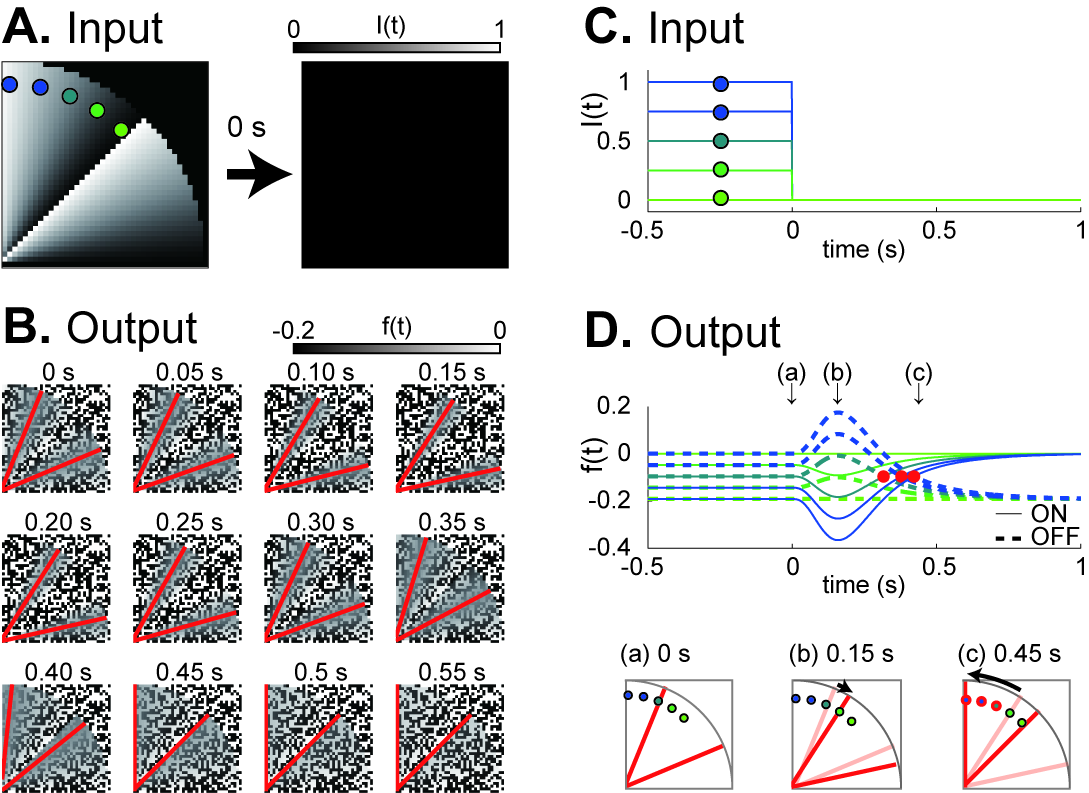

Supplement: S2 Figure — The retinal ON/OFF model produced an afterimage rotation of the FW stimulus on the dark background. (A) Input. One quarter of the FW stimulus suddenly disappeared and was replaced by the dark background at 0 s. (B) Outputs after the disappearance of the FW stimulus. Red lines indicate the focus lines on which the ON- and OFF-type responses, fON(t) and fOFF(t) in Fig. 2, respectively, showed comparable values. (C) Time courses of inputs. Each marked line corresponds to the input time-series (in terms of the luminance level) at the marked position in (A). (D) Time courses of outputs from the ON- and OFF-type units (top). Each colored line corresponds to the output in response to the input with the same color in (C). Red lines and points indicate the focus lines and points. Schematic drawing of outputs at 0 s (a), 0.15 s (b) and 0.45 s (c) (bottom). Focus lines slightly rotated counterclockwise from 0 s (thin red lines) to 0.15 s (red lines) (b), then prominently rotated clockwise from 0.15 s (thin red lines) to 0.45 s (red lines) (c). The red marked points in (c) correspond to the three red points in the top panel. (TIF) [file pone.0115464.s002.tif]

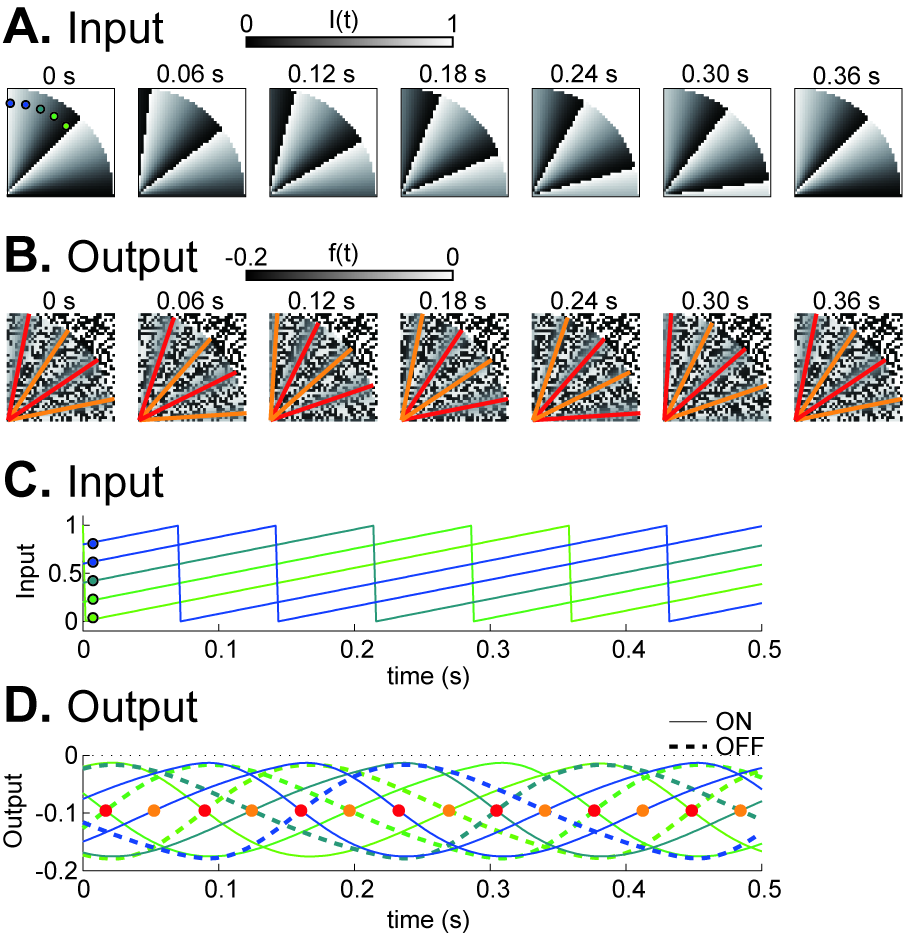

Supplement: S3 Figure — Focus rotation of ON- and OFF-type responses compared with actual rotation of the FW stimulus. (A) Input. The FW stimulus rotated clockwise with an angular velocity of 125°/s. (B) Output. Red and orange lines indicate focus lines on both of which the ON- and OFF-type responses showed comparable values. The number of focus lines was doubled from that in Fig. 2, because the focus line rotation appeared not only at the timing of the disappearance, but also at the timing of the appearance of the FW stimulus. Note that the appearance and disappearance occurred at each location, suggesting that it was elicited by the actually rotating FW stimulus. (C) Time courses of inputs. Each marked line corresponds to the input time-series at the marked position in (A). (D) Time courses of outputs from the ON- and OFF-type units. Each colored line represents the output in response to the input with the same color as in (C). Red and orange points indicate focus points. (TIF) [file pone.0115464.s003.tif]

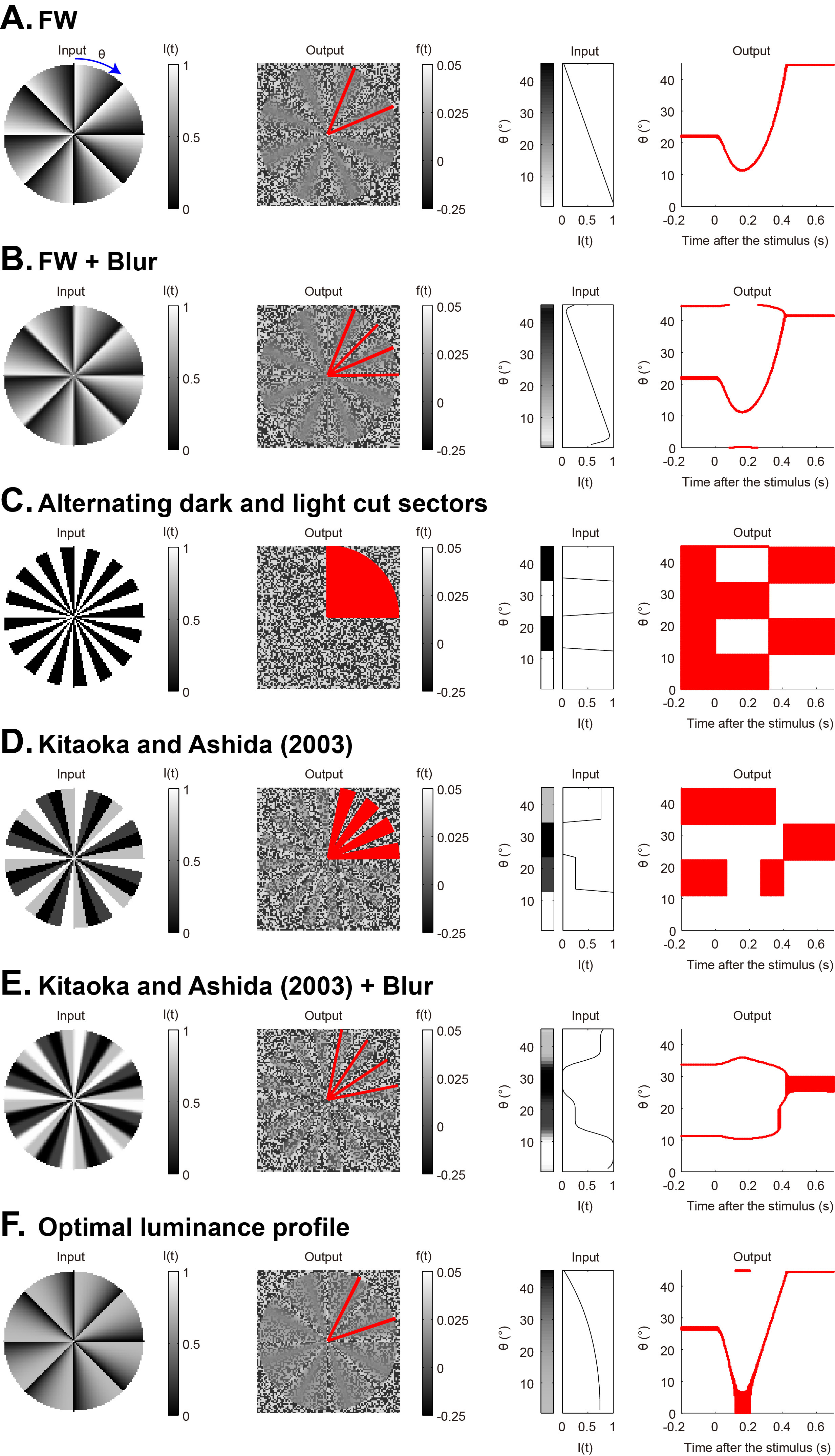

Supplement: S4 Figure — Focus line rotation of ON- and OFF-type responses to various types of visual stimuli: (A) the FW stimulus, (B) the blurred FW stimulus (Gaussian filter: σ = 1.35°), (C) alternating dark and light cut sectors, (D) the Kitaoka–Ashida stimulus [22], (E) the blurred Kitaoka–Ashida stimulus (Gaussian filter: σ = 1.35°), and (F) the optimal luminance profile. The first column shows the input image on the light background. The second column shows the output from the retinal ON/OFF model at 0 s where the red lines indicate the focus lines of the ON- and OFF-type responses (just for the top right quarter of the whole output). The third column shows the luminance profile of the input image, and the fourth column shows the time development of the angles of the focus lines (red lines). (JPG) [file pone.0115464.s004.jpg]
