# Supplementary material for: A Computational Model of Afterimage Rotation in the Peripheral Drift Illusion Based on Retinal ON/OFF Responses
Source: PLoS One. 2014 Dec 17;9(12):e115464. doi: 10.1371/journal.pone.0115464 (PMC4269430; doi:10.1371/journal.pone.0115464)
Supplement: S1 Table — Data points of Fig. 4B and results of experiment 1. Probability of seeing clockwise rotation by the three observers (Y.H., S.S., and R.O.). In each trial, the background luminance was randomly selected from 11 linearly-spaced intensities. (PDF) [file pone.0115464.s005.pdf]

Data points of Fig. 4B

Y.H.

Relative luminance	0.00	0.10	0.20	0.30	0.40	0.50	0.60	0.70	0.80	0.90	1.00
Probability of judgment "Clockwise"	8.33	33.33	25.00	41.67	83.33	100.00	83.33	100.00	100.00	100.00	100.00

S.S.

Relative luminance	0.00	0.10	0.20	0.30	0.40	0.50	0.60	0.70	0.80	0.90	1.00
Probability of judgment "Clockwise"	41.67	16.67	16.67	25.00	41.67	66.67	66.67	91.67	91.67	100.00	91.67

R.O.

Relative luminance	0.00	0.10	0.20	0.30	0.40	0.50	0.60	0.70	0.80	0.90	1.00
Probability of judgment "Clockwise"	25.00	58.33	25.00	33.33	91.67	91.67	100.00	91.67	100.00	100.00	100.00

Results of experiment 1 (1)

Left or right: Side of presentation of the FW stimulus (left, 1; right, 0)
Back. lum.: Background luminance ranging from 0 to 255 (dark to light)
Judge: Judgment of the participant (clockwise, 1; counterclockwise, -1)

Participant: Y.H.

Trial No.	Left or right	Back. lum.	Judge	Trial No.	Left or right	Back. lum.	Judge	Trial No.	Left or right	Back. lum.	Judge
1	0	255	1	45	1	77	-1	89	0	0	-1
2	1	204	1	46	1	26	-1	90	0	0	-1
3	1	255	1	47	1	128	1	91	0	153	-1
4	1	77	-1	48	0	51	-1	92	1	26	1
5	1	153	1	49	0	51	-1	93	0	179	1
6	0	102	1	50	0	230	1	94	1	51	1
7	0	128	1	51	1	255	1	95	0	102	1
8	0	0	-1	52	0	179	1	96	1	179	1
9	1	51	-1	53	0	204	1	97	0	51	-1
10	1	128	1	54	1	77	1	98	1	255	1
11	1	153	1	55	1	51	1	99	1	230	1
12	1	102	1	56	1	230	1	100	0	255	1
13	1	102	1	57	0	26	-1	101	0	0	-1
14	0	26	-1	58	1	0	-1	102	1	255	1
15	0	77	-1	59	0	102	1	103	0	153	-1
16	1	0	-1	60	1	153	1	104	0	26	-1
17	1	26	-1	61	0	0	1	105	1	179	1
18	1	51	-1	62	1	179	1	106	1	153	1
19	1	77	-1	63	0	230	1	107	1	77	-1
20	0	255	1	64	0	230	1	108	1	255	1
21	1	204	1	65	0	77	1	109	0	102	1
22	1	0	-1	66	0	128	1	110	0	204	1
23	0	179	1	67	0	102	1	111	0	26	1
24	1	204	1	68	1	26	1	112	0	102	1
25	1	0	-1	69	1	77	-1	113	0	77	1
26	0	153	1	70	1	102	1	114	1	179	1
27	1	51	-1	71	0	102	-1	115	0	77	1
28	1	179	1	72	1	0	-1	116	0	153	1
29	1	230	1	73	0	153	1	117	1	128	1
30	0	102	-1	74	1	0	-1	118	0	128	1
31	0	179	1	75	0	179	1	119	0	204	1
32	1	230	1	76	1	255	1	120	0	230	1
33	0	204	1	77	1	51	-1	121	0	26	-1
34	0	153	1	78	1	128	1	122	0	230	1
35	0	255	1	79	0	255	1	123	1	204	1
36	0	128	1	80	1	255	1	124	1	77	1
37	1	128	1	81	1	153	1	125	1	0	-1
38	0	230	1	82	0	128	1	126	1	204	1
39	1	77	-1	83	0	26	-1	127	0	230	1
40	1	51	-1	84	0	179	1	128	1	102	1
41	1	26	1	85	0	204	1	129	1	128	1
42	0	230	1	86	1	153	1	130	1	51	-1
43	0	26	-1	87	0	204	1	131	1	128	1
44	0	179	1	88	0	204	1	132	1	51	1

Results of experiment 1 (2)

Left or right: Side of presentation of the FW stimulus (left, 1; right, 0)
Back. lum.: Background luminance ranging from 0 to 255 (dark to light)
Judge: Judgment of the participant (clockwise, 1; counterclockwise, -1)

Participant: S.S.

Trial No.	Left or right	Back. lum.	Judge	Trial No.	Left or right	Back. lum.	Judge	Trial No.	Left or right	Back. lum.	Judge
1	0	0	1	45	1	204	1	89	1	77	-1
2	0	0	-1	46	1	0	1	90	1	26	-1
3	0	153	-1	47	0	179	1	91	1	128	1
4	1	26	1	48	0	102	1	92	0	51	-1
5	0	179	1	49	1	230	1	93	0	51	-1
6	1	51	-1	50	0	128	-1	94	0	230	1
7	0	102	-1	51	0	77	-1	95	1	255	1
8	1	179	1	52	1	255	1	96	0	179	-1
9	0	51	-1	53	0	255	1	97	0	204	1
10	1	255	1	54	1	26	-1	98	1	77	-1
11	1	230	1	55	0	102	1	99	1	51	-1
12	0	255	-1	56	1	77	-1	100	1	230	1
13	0	0	-1	57	1	0	-1	101	0	26	-1
14	1	255	1	58	1	255	1	102	1	0	-1
15	0	153	1	59	1	179	1	103	0	102	-1
16	0	26	1	60	0	128	1	104	1	153	-1
17	1	179	1	61	1	153	1	105	0	0	-1
18	1	153	1	62	0	230	1	106	1	179	1
19	1	77	1	63	1	102	-1	107	0	230	1
20	1	255	1	64	1	255	1	108	0	230	1
21	0	102	-1	65	1	77	-1	109	0	77	-1
22	0	204	1	66	0	51	-1	110	0	128	1
23	0	26	-1	67	1	230	1	111	0	102	-1
24	0	102	-1	68	1	51	1	112	1	26	-1
25	0	77	1	69	1	77	-1	113	1	77	-1
26	1	179	1	70	0	179	1	114	1	102	1
27	0	77	-1	71	1	128	-1	115	0	102	1
28	0	153	-1	72	0	230	1	116	1	0	1
29	1	128	1	73	1	128	1	117	0	153	1
30	0	128	-1	74	0	26	-1	118	1	0	-1
31	0	204	1	75	1	204	1	119	0	179	1
32	0	230	1	76	0	179	1	120	1	255	1
33	0	26	-1	77	1	0	-1	121	1	51	-1
34	0	230	1	78	1	26	-1	122	1	128	1
35	1	204	1	79	1	0	1	123	0	255	1
36	1	77	1	80	0	204	1	124	1	255	1
37	1	0	1	81	0	204	1	125	1	153	1
38	1	204	-1	82	0	153	-1	126	0	128	-1
39	0	230	1	83	1	102	-1	127	0	26	-1
40	1	102	1	84	0	153	1	128	0	179	1
41	1	128	1	85	0	51	-1	129	0	204	1
42	1	51	1	86	0	51	-1	130	1	153	1
43	1	128	1	87	0	26	-1	131	0	204	1
44	1	51	-1	88	0	153	1	132	0	204	1

Results of experiment 1 (3)

Left or right: Side of presentation of the FW stimulus (left, 1; right, 0)
Back. lum.: Background luminance ranging from 0 to 255 (dark to light)
Judge: Judgment of the participant (clockwise, 1; counterclockwise, -1)

Participant: R.O.

Trial No.	Left or right	Back. lum.	Judge	Trial No.	Left or right	Back. lum.	Judge	Trial No.	Left or right	Back. lum.	Judge
1	1	204	1	45	1	77	-1	89	0	0	-1
2	1	0	-1	46	1	26	1	90	0	0	1
3	0	179	1	47	1	128	1	91	0	153	1
4	0	102	1	48	0	51	-1	92	1	26	-1
5	1	230	1	49	0	51	-1	93	0	179	1
6	0	128	1	50	0	230	1	94	1	51	-1
7	0	77	1	51	1	255	1	95	0	102	1
8	1	255	1	52	0	179	1	96	1	179	1
9	0	255	1	53	0	204	1	97	0	51	-1
10	1	26	1	54	1	77	-1	98	1	255	1
11	0	102	1	55	1	51	-1	99	1	230	1
12	1	77	1	56	1	230	1	100	0	255	1
13	1	0	-1	57	0	26	1	101	0	0	-1
14	1	255	1	58	1	0	-1	102	1	255	1
15	1	179	-1	59	0	102	1	103	0	153	1
16	0	128	1	60	1	153	1	104	0	26	-1
17	1	153	1	61	0	0	-1	105	1	179	1
18	0	230	1	62	1	179	1	106	1	153	1
19	1	102	1	63	0	230	1	107	1	77	-1
20	1	255	1	64	0	230	1	108	1	255	1
21	1	77	-1	65	0	77	-1	109	0	102	1
22	0	51	-1	66	0	128	1	110	0	204	1
23	1	230	1	67	0	102	1	111	0	26	-1
24	1	51	-1	68	1	26	1	112	0	102	1
25	1	77	1	69	1	77	-1	113	0	77	-1
26	0	179	1	70	1	102	1	114	1	179	1
27	1	128	1	71	0	102	1	115	0	77	1
28	0	230	1	72	1	0	1	116	0	153	1
29	1	128	1	73	0	153	1	117	1	128	1
30	0	26	1	74	1	0	-1	118	0	128	-1
31	1	204	1	75	0	179	1	119	0	204	1
32	0	179	1	76	1	255	1	120	0	230	1
33	1	0	-1	77	1	51	1	121	0	26	1
34	1	26	-1	78	1	128	1	122	0	230	1
35	1	0	1	79	0	255	1	123	1	204	1
36	0	204	1	80	1	255	1	124	1	77	-1
37	0	204	1	81	1	153	1	125	1	0	-1
38	0	153	1	82	0	128	1	126	1	204	1
39	1	102	-1	83	0	26	-1	127	0	230	1
40	0	153	1	84	0	179	1	128	1	102	1
41	0	51	1	85	0	204	1	129	1	128	1
42	0	51	1	86	1	153	1	130	1	51	-1
43	0	26	1	87	0	204	1	131	1	128	1
44	0	153	1	88	0	204	1	132	1	51	-1
