# Supplementary material for: A Computational Model of Afterimage Rotation in the Peripheral Drift Illusion Based on Retinal ON/OFF Responses
Source: PLoS One. 2014 Dec 17;9(12):e115464. doi: 10.1371/journal.pone.0115464 (PMC4269430; doi:10.1371/journal.pone.0115464)
Supplement: S2 Table — Data points of Fig. 5B and results of experiment 2. Probability of seeing slower afterimage rotation than the reference rotation by the three observers (Y.H., Y.A., and T.M.). In each trial, the rotation speed of the rotating FW stimulus was randomly selected from nine predefined parameters (11.3°/s, 22.5°/s, 45°/s, 56.3°/s, 67.5°/s, 112.5°/s, 168.8°/s, 225°/s, 337.5°/s). (PDF) [file pone.0115464.s006.pdf]

Data points of Fig. 5B

Y.H.

Rotation velocity (°/s)	11.30	22.50	45.00	56.30	67.50	112.50	168.80	225.00	337.50
Probability of judgment "Slow illusion"	0.00	0.00	0.00	16.67	33.33	50.00	75.00	91.67	100.00

Y.A.

Rotation velocity (°/s)	11.30	22.50	45.00	56.30	67.50	112.50	168.80	225.00	337.50
Probability of judgment "Slow illusion"	0.00	0.00	0.00	0.00	8.33	25.00	66.67	91.67	100.00

T.M.

Rotation velocity (°/s)	11.30	22.50	45.00	56.30	67.50	112.50	168.80	225.00	337.50
Probability of judgment "Slow illusion"	0.00	0.00	16.67	16.67	16.67	33.33	58.33	91.67	91.67

Results of experiment 2 (1)

Left or right: Side of presentation of the still image (left, 1; right, 0)
Rot. speed: Rotation speed of the rotating FW stimulus
Judge: Judgment which stimulus rotated more rapidly (seemingly rotating, 1; actually rotating, -1)

Participant: Y.H.

Trial No.	Left or right	Rot. speed	Judge	Trial No.	Left or right	Rot. speed	Judge	Trial No.	Left or right	Rot. speed	Judge
1	1	22.5	1	37	0	112.5	1	73	1	45	1
2	0	56.3	-1	38	0	168.8	-1	74	0	45	-1
3	1	11.3	1	39	0	337.5	1	75	0	168.8	1
4	0	11.3	-1	40	0	67.5	-1	76	1	67.5	1
5	0	225	1	41	0	112.5	-1	77	1	337.5	-1
6	0	337.5	1	42	0	45	-1	78	0	225	1
7	1	112.5	1	43	0	22.5	-1	79	0	67.5	-1
8	1	168.8	1	44	0	45	-1	80	1	337.5	-1
9	0	22.5	-1	45	1	225	-1	81	0	337.5	1
10	0	11.3	-1	46	1	45	1	82	1	11.3	1
11	0	225	1	47	0	225	1	83	1	112.5	1
12	1	45	1	48	0	56.3	-1	84	1	112.5	-1
13	1	67.5	1	49	0	45	-1	85	0	45	-1
14	1	112.5	-1	50	1	11.3	1	86	0	56.3	-1
15	0	225	1	51	0	67.5	-1	87	0	225	1
16	1	225	-1	52	0	56.3	-1	88	1	56.3	1
17	1	22.5	1	53	1	168.8	-1	89	0	112.5	1
18	0	56.3	-1	54	0	337.5	1	90	1	56.3	1
19	0	45	-1	55	0	11.3	-1	91	0	225	1
20	0	22.5	-1	56	0	168.8	1	92	1	337.5	-1
21	1	45	1	57	0	22.5	-1	93	0	168.8	1
22	0	45	-1	58	0	22.5	-1	94	1	11.3	1
23	1	67.5	-1	59	1	11.3	1	95	1	22.5	1
24	0	112.5	1	60	1	337.5	-1	96	1	56.3	-1
25	1	337.5	-1	61	1	337.5	-1	97	1	11.3	1
26	0	11.3	-1	62	0	112.5	-1	98	0	22.5	-1
27	1	56.3	1	63	0	56.3	-1	99	1	112.5	1
28	1	337.5	-1	64	0	67.5	1	100	0	67.5	-1
29	1	337.5	-1	65	1	225	-1	101	0	67.5	1
30	1	67.5	1	66	0	11.3	-1	102	1	22.5	1
31	1	56.3	1	67	0	67.5	1	103	1	45	1
32	0	112.5	1	68	0	22.5	-1	104	0	168.8	1
33	0	168.8	1	69	1	56.3	-1	105	1	225	-1
34	0	67.5	-1	70	1	225	1	106	1	22.5	1
35	1	168.8	-1	71	1	112.5	1	107	1	11.3	1
36	0	168.8	-1	72	0	168.8	1	108	1	168.8	-1

Results of experiment 2 (2)

Left or right: Side of presentation of the still image (left, 1; right, 0)
Rot. speed: Rotation speed of the rotating FW stimulus
Judge: Judgment which stimulus rotated more rapidly (seemingly rotating, 1; actually rotating, -1)

Participant: Y.A.

Trial No.	Left or right	Rot. speed	Judge	Trial No.	Left or right	Rot. speed	Judge	Trial No.	Left or right	Rot. speed	Judge
1	1	112.5	1	37	0	56.3	-1	73	0	112.5	-1
2	0	45	-1	38	0	45	-1	74	1	67.5	1
3	0	22.5	-1	39	0	22.5	-1	75	1	112.5	-1
4	1	56.3	1	40	0	67.5	-1	76	1	45	1
5	1	168.8	-1	41	0	67.5	-1	77	1	56.3	1
6	0	67.5	-1	42	0	112.5	1	78	1	168.8	-1
7	0	67.5	-1	43	0	112.5	-1	79	0	67.5	-1
8	0	112.5	-1	44	1	11.3	1	80	1	22.5	1
9	0	67.5	-1	45	1	45	1	81	0	11.3	-1
10	0	22.5	-1	46	1	11.3	1	82	1	168.8	1
11	0	337.5	1	47	1	45	1	83	1	56.3	1
12	1	11.3	1	48	1	112.5	1	84	1	22.5	1
13	0	56.3	-1	49	0	56.3	-1	85	1	112.5	1
14	1	67.5	1	50	0	337.5	1	86	1	337.5	-1
15	1	56.3	1	51	1	11.3	1	87	1	67.5	1
16	1	22.5	1	52	0	22.5	-1	88	1	11.3	1
17	1	168.8	1	53	1	11.3	1	89	1	112.5	-1
18	1	112.5	1	54	1	337.5	-1	90	1	22.5	1
19	0	56.3	-1	55	1	112.5	1	91	0	337.5	1
20	1	45	1	56	0	67.5	1	92	0	11.3	-1
21	0	22.5	-1	57	0	337.5	1	93	1	168.8	-1
22	1	45	1	58	1	168.8	-1	94	0	22.5	-1
23	1	45	1	59	1	67.5	1	95	1	11.3	1
24	0	11.3	-1	60	1	56.3	1	96	0	337.5	1
25	0	337.5	1	61	0	56.3	-1	97	1	225	-1
26	0	112.5	-1	62	0	45	-1	98	0	225	1
27	1	11.3	1	63	1	22.5	1	99	1	225	1
28	1	337.5	-1	64	0	337.5	1	100	0	225	1
29	0	168.8	1	65	0	45	-1	101	1	225	-1
30	1	168.8	1	66	1	67.5	1	102	0	225	1
31	1	11.3	1	67	1	56.3	1	103	1	225	-1
32	0	337.5	1	68	1	56.3	1	104	1	225	-1
33	1	22.5	1	69	1	45	1	105	0	225	1
34	1	168.8	1	70	0	168.8	1	106	1	225	-1
35	1	168.8	-1	71	1	337.5	-1	107	1	225	-1
36	1	168.8	-1	72	0	45	-1	108	0	225	1

Results of experiment 2 (3)

Left or right: Side of presentation of the still image (left, 1; right, 0)
Rot. speed: Rotation speed of the rotating FW stimulus
Judge: Judgment which stimulus rotated more rapidly (seemingly rotating, 1; actually rotating, -1)

Participant: T.M.

Trial No.	Left or right	Rot. speed	Judge	Trial No.	Left or right	Rot. speed	Judge	Trial No.	Left or right	Rot. speed	Judge
1	0	67.5	1	37	1	112.5	1	73	1	225	-1
2	1	112.5	1	38	0	225	1	74	1	22.5	1
3	0	45	-1	39	1	337.5	-1	75	1	11.3	1
4	1	225	1	40	1	67.5	1	76	1	56.3	1
5	0	22.5	-1	41	1	22.5	1	77	0	45	1
6	0	22.5	-1	42	1	225	-1	78	0	337.5	1
7	0	11.3	-1	43	1	337.5	-1	79	0	112.5	-1
8	1	168.8	-1	44	0	67.5	-1	80	0	45	-1
9	1	337.5	-1	45	0	11.3	-1	81	1	337.5	-1
10	1	168.8	1	46	0	67.5	-1	82	0	45	-1
11	0	225	1	47	1	337.5	1	83	0	337.5	1
12	1	45	1	48	1	168.8	1	84	0	337.5	1
13	1	11.3	1	49	1	22.5	1	85	0	56.3	-1
14	1	112.5	1	50	0	56.3	1	86	1	67.5	1
15	1	67.5	1	51	1	168.8	1	87	0	67.5	-1
16	1	56.3	1	52	0	45	-1	88	1	225	-1
17	0	56.3	-1	53	0	67.5	1	89	1	56.3	1
18	1	56.3	1	54	0	56.3	1	90	1	168.8	1
19	0	45	1	55	1	11.3	1	91	0	112.5	-1
20	0	22.5	-1	56	0	112.5	1	92	0	168.8	1
21	1	67.5	1	57	0	225	1	93	0	168.8	1
22	0	11.3	-1	58	0	337.5	1	94	0	225	1
23	0	337.5	1	59	0	11.3	-1	95	1	67.5	1
24	1	168.8	-1	60	0	112.5	1	96	0	22.5	-1
25	1	225	-1	61	1	168.8	-1	97	1	45	1
26	0	112.5	1	62	1	112.5	1	98	0	225	1
27	1	337.5	-1	63	1	168.8	-1	99	1	67.5	1
28	0	225	1	64	1	225	-1	100	1	168.8	1
29	1	22.5	1	65	1	45	1	101	1	22.5	1
30	1	11.3	1	66	1	45	1	102	1	11.3	1
31	0	56.3	-1	67	1	22.5	1	103	0	112.5	1
32	1	112.5	1	68	1	45	1	104	0	11.3	-1
33	1	45	1	69	0	56.3	-1	105	0	112.5	-1
34	1	168.8	-1	70	1	11.3	1	106	1	22.5	1
35	1	67.5	1	71	1	56.3	1	107	1	11.3	1
36	0	337.5	1	72	1	22.5	1	108	0	56.3	-1
